# KiGGS Wave 2 cross-sectional study – participant acquisition, response rates and representativeness

**DOI:** 10.17886/RKI-GBE-2018-032

**Published:** 2018-03-15

**Authors:** Robert Hoffmann, Michael Lange, Hans Butschalowsky, Robin Houben, Patrick Schmich, Jennifer Allen, Ronny Kuhnert, Angelika Schaffrath Rosario, Antje Gößwald

**Affiliations:** Robert Koch Institute, Berlin, Department of Epidemiology and Health Monitoring

**Keywords:** RESPONSE RATE, REPRESENTATIVENESS, KIGGS, CHILDREN AND ADOLESCENTS, HEALTH MONITORING

## Abstract

For the third time, wave 2 of the German Health Interview and Examination Survey for Children and Adolescents (KiGGS), which is conducted in the context of health monitoring at the Robert Koch Institute, now provides representative cross-sectional data for Germany. Completed in 2017, data for the cross-sectional component of KiGGS Wave 2 was collected in the form of an interview and examination survey. Interview survey data was collected from 15,023 participants, meaning that the required number of participants has been reached. A randomly selected subgroup of 3,567 participants was also examined. The overall response rate was 40.1%. Differences in response rates were registered regarding certain sociodemographic characteristics. Weighting was applied to compensate for differences in willingness to participate related to age, gender, geographic region, nationality and education factors. Weighting ensures that assessments of the health of children and adolescents in Germany are representative for the population. The data serves to estimate prevalence rates and, through comparison with the results from previous survey waves, to analyse trends. A set of measures were taken to recruit a sufficiently large group of participants and ensure that the net sample reflects the composition of the overall population to the highest degree. For future surveys, further measures ought to be taken in order to improve the integration of hard-to-reach subgroups.

## 1. Background

**Figure 1:**
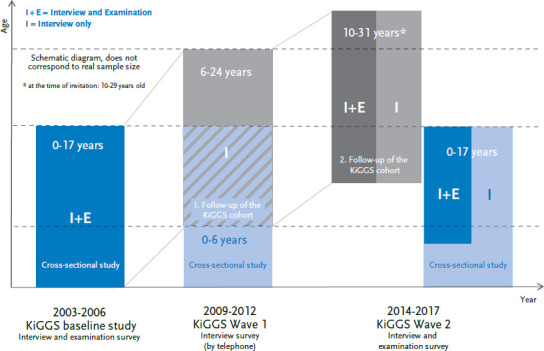
KiGGS study design Source: Based on Mauz et al. [4]

The German Health Interview and Examination Survey for Children and Adolescents (KiGGS) forms part of the health monitoring at the Robert Koch Institute (RKI) [1, 2]. An important goal of KiGGS is to regularly provide reliable information on the health, health behaviour and utilisation of health care services by children and adolescents aged 0 to 17 years in Germany. With the completion of KiGGS Wave 2 in 2017, the RKI now has, following the KiGGS baseline study (2003-2006) and KiGGS Wave 1 (2009-2012), for the third time collected up-to-date cross-sectional data on the health of children and adolescents in Germany. Based on this data, the RKI can estimate prevalence rates for the surveyed indicators and – by comparing these with those surveyed in previous waves – identify trends. Moreover, KiGGS, by returning to study participants in the baseline study (2003-2006) [3], contains a longitudinal component (KiGGS cohort) that serves to analyse longitudinal relationships and describe individual level developments [4]. Lange et al [5] presents this longitudinal data from KiGGS Wave 2 in this issue. Figure 1 illustrates the KiGGS study design.

Whereas KiGGS Wave 1 was conducted purely as a telephone interview survey, KiGGS Wave 2 (like the baseline study) comprised an interview and examination component. Surveying in KiGGS Wave 2 again included collecting data on physical and mental health, health behaviour, utilisation of health care services and prevention as well as data on social, family and environmental factors. In KiGGS Wave 2, interviews were conducted with all participants. In addition, for a randomly selected subgroup, physical examinations, tests and laboratory analyses of blood and urine samples were conducted [6]. As in previous KiGGS surveys, surveying instruments were differentiated according to age groups [7]. A detailed description of the methodology can be found in New data for action. Data collection for KiGGS Wave 2 has been completed [4]. A set of independent modules flanks the core survey and collected specific data on determined questions in subsamples. KiGGS survey data is used in federal health reporting, epidemiological analyses as well as public health research. Moreover, the data is made available to researchers (via public use files) [4]. The generated results are an important basis of information for health policy, health care and prevention stakeholders [2].

**KiGGS Wave 2**

Second follow-up to the German Health Interview and Examination Survey for Children and Adolescents

**Data owner:** Robert Koch Institute

**Aim:** Providing reliable information on health status, health-related behaviour, living conditions, protective and risk factors, and health care among children, adolescents and young adults living in Germany, with the possibility of trend and longitudinal analyses

**Study design**: Combined cross-sectional and cohort study

**Cross-sectional study in KiGGS Wave 2**

**Age range:** 0-17 years

**Population:** Children and adolescents with permanent residence in Germany

**Sampling:** Samples from official residency registries - randomly selected children and adolescents from the 167 cities and municipalities covered by the KiGGS baseline study

**Sample size:** 15,023 participants

**KiGGS cohort study in KiGGS Wave 2**

**Age range:** 10-31 years

**Sampling:** Re-invitation of everyone who took part in the KiGGS baseline study and who was willing to participate in a follow-up

**Sample size:** 10,853 participants

**KiGGS survey waves**

► KiGGS baseline study (2003-2006), examination and interview survey
► KiGGS Wave 1 (2009-2012), interview survey
► KiGGS Wave 2 (2014-2017), examination and interview survey

More information is available at www.kiggs-studie.de/english

To reliably estimate prevalence rates a cross-sectional sample is required. It needs to be both sufficiently large and also needs to representatively reflect the composition of the target population – in this case all children and adolescents aged under 18 with permanent residence in Germany.

The aim of this article is to enhance the assessment of the data published in this issue and future publications of cross-sectional results from KiGGS Wave 2. Initially, we present the sampling method used and the measures taken to recruit participants. Subsequently, we present the response rates that were achieved and how the composition of the realised sample was controlled. This is followed by a description of how we developed weighting factors, which were then applied to compensate for the different levels of participation between surveyed subgroups. To conclude, we discuss the approach and provide an outlook on further planned analyses.

## 2. Methodology

### 2.1 Sample

The survey estimates prevalence rates for children and adolescents aged 0 to 17 years with permanent residence in Germany. To represent this population, a sampling process involving two steps was applied:

► In a first step, sample points were selected. The survey used the 167 sample points that were selected in co-operation with GESIS (Leibniz Institute for the Social Sciences, formerly the ZUMA) for the KiGGS baseline study [8]. The selection procedure ensures that sample points reflect Germany’s regional structure regarding federal state and type of municipality (BIK classification [9]). The validity of these sample points was re-assessed for KiGGS Wave 2. Drawing of points was biased towards municipalities in former East Germany to produce more precise information on this region.
► The second step involved randomly selecting addresses of children and adolescents from the corresponding municipal population registries for each sample point. To achieve the stipulated same number of cases from all sample points, a different number of addresses was drawn for each age cohort depending on size of municipality, region and the response rates that were achieved in the KiGGS baseline study. An oversampling factor of 1.5 was applied to children and adolescents, who do not hold German nationality to compensate for the expected higher share of quality neutral losses and the lower response rates of this segment of the population [8]. Participants who do not belong to the target population are defined as quality neutral losses (see chapter 2.6).

Once the addresses of selected children and adolescents – hereafter referred to as study participants – had been received from the corresponding population registries, they were randomly divided into two groups at the Robert Koch Institute. Participants in the first group covering the total age range from 0 to 17 years were invited only to the interview component (interview group). Those in the second group in the age range 3 to 17 years were also invited to take part in the examination component (called the examination and interview group in the following). Concerning the number of cases (net sample), the goal was to recruit 9,000 children and adolescents for the interview group and 3,750 for the combined examination and interview group. Hence, the plan was to collect interview data from a total of 12,750 participants. Both groups were kept strictly separated during the entire process from invitation to data collection. Study participants could not switch between the two groups.

### 2.2 Data collection process

Cross-sectional data collection in KiGGS Wave 2 was conducted between September 2014 and June 2017. The parents of all study participants received a child health questionnaire and an additional health questionnaire for children and adolescents aged at least eleven years. They also received a questionnaire on dietary habits to be filled out by the parents, or by the children and adolescents themselves when aged at least eleven. As a pilot study neither indicated an increase in willingness to participate nor a positive effect on sample composition in a target population aged under 18 through the additional use of online questionnaires, the survey exclusively used self-administered paper questionnaires [10].

Examinations were conducted at examination centres created specifically for this reason at the sample points over an eight-day period. Examination staff teams worked in parallel, meaning that examinations were conducted simultaneously at three locations. The order in which sample points were visited was systematically scheduled in a so-called road map to reduce the effect of seasonal or regional factors [11].

### 2.3 Invitation and recruiting of participants

**Figure 2:**
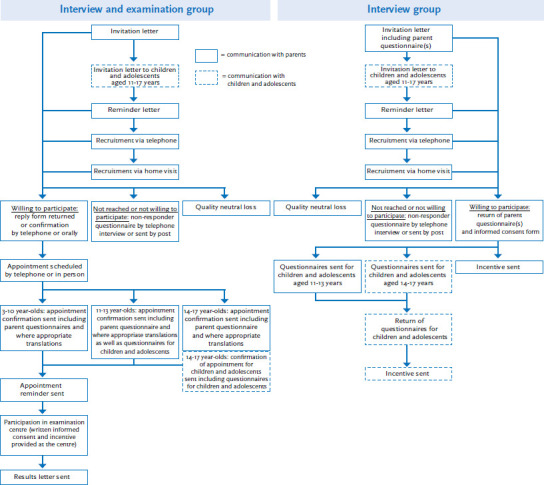
Participant recruiting in the interview and examination group and the interview group Source: Own diagram

Invitations were sent for both groups simultaneously based on the schedule laid out by the road map. Invitation letters (Figure 2) were usually sent to parents or legal guardians (for reasons of simplification called parents below) six weeks before the opening of an examination centre at a particular sample point. As the study participants were minors, for legal and ethical reasons, parents were the contact persons in the survey. Invitation letters included a comprehensive information brochure that gave details about the survey institution, survey content and data protection. Usually three days after the invitation letter, children and adolescents, who were aged at least eleven years, received their own letter of invitation, providing them with information, which was adapted specifically for this age group. These letters were followed by three further steps to increase the number of participants, in an attempt to reach as many of those parents, who had failed to answer the invitation. First, about ten days after the initial invitation, parents were sent a reminder. Second, about two weeks later, parents were contacted by telephone to convince them to participate. Telephone numbers were researched through commercially available telephone number registries. This was carried out because the number of available telephone numbers was limited, as many households no longer have a landline and the telephone numbers of many people are no longer registered in telephone books [12]. If it was not possible to find out a person’s telephone number (or call a person), the third measure consisted of visiting parents during the week before the examination centre opened its doors. Contacting potential participants by telephone and, in particular, visiting them at their homes prior to the survey, proved an important step in recruiting participants. One-to-one conversations can help dispel reservations, close information gaps and increase trust in the survey goals and the integrity of the survey institution. Specially trained staff was employed in these recruitment measures.

### 2.4 Further measures to increase participation

Numerous measures aimed to increase participation in the cross-sectional and longitudinal components of KiGGS Wave 2 with regard to size and composition of the realised sample. Lange et al. [5] describes the specific measures applied in the longitudinal component.

► **Information management:** All of the information material used during survey implementation was developed to ensure it conveys the relevant information for each of the specific target groups. The focus was on preparing the information in a way that it is adequate for the different target groups, easily understandable and visually appealing. Potential participants could also access further information from the survey website (www.kiggs-studie.de). Furthermore, a free phone number was provided, and email contact in case of any questions was also always possible. Local public relations efforts at each sample point were processed and aimed to ensure coverage of the survey in local media and the spreading of information.
► **Incentives to participate:** A set of incentives was featured in the information material and during telephone calls and personal contact. Participants in the interview component were offered a shopping voucher. People, who took part in the examination component, were offered non-monetary gifts, cash, as well as a personal results report including laboratory test results depending on the age of the study participant.
► **Reducing barriers to participation:** This included the provision of self-addressed envelopes marked “postage to be paid by recipient” and aimed to make it easier for participants to return the forms, questionnaires and consent forms (Figure 2).
► **Appointment management in the examination component:** Appointments were scheduled to take account of the limited time of parents, children and adolescents. Appointments were therefore also possible either in the early morning or in the early evening. Appointments on Saturdays were also possible. People who were willing to participate, yet for whom no (fitting) appointment could be made, were put on a waiting list to be contacted at short notice by telephone in case an appointment at another time became vacant. Examinations were scheduled to take approximately two hours depending on the survey programme (differentiated by age). To reduce the amount of time participants spent at the examination centres, they received the questionnaires in advance (Figure 2), and were asked to fill them out at home beforehand.
► **Measures for migrants:** Finally, there was a set of measures aimed to improve participation in the survey of people with migration background. They are described in detail in Frank et al. [13] in this issue and included translated invitation letters, questionnaires and consent forms. Moreover, the entire survey staff received culture-sensitive communication training.

### 2.5 Non-responder follow-up

The children of parents, who were unable or unwilling to participate in the survey, were asked about the reasons for their decision and, subsequently, to fill out a short questionnaire. This questionnaire comprised questions on health and health behaviour as well as socio-demographic characteristics that were also surveyed for participants. Based on this information, it becomes possible to compare non-responders and participants against key indicators and potentially determine systematic differences between the two groups.

### 2.6 Response rate calculation

Study participants were excluded from the gross sample as quality neutral losses when they did not belong to the target population. Reasons for this classification were majority age, decease, twofold drawn, invalid addresses (invitation was undeliverable, address outside the sample point) or moved to a foreign country. Another reason was the impediment of adequate communication with parents through language barriers. These exclusions were necessary in order to ensure sufficient actual information of the parents about the survey and the examination programme for ethical and medical reasons.

Response rates are calculated according to AAPOR Response Rate 2 [14]. It is the number of participants divided by the number of gross sample members reduced by quality neutral losses.

### 2.7 Data protection and ethics

KiGGS Wave 2 is subject to strict compliance with the data protection provisions set out in the Federal Data Protection Act. Hannover Medical School’s ethics committee examined and approved the ethics of the study (No. 2275-2014). The Federal Commissioner for Data Protection and Freedom of Information in Germany received the KiGGS Wave 2 study concept and had no objections. Together with the invitation to the survey, participants, their parents and/or legal guardians were informed about those responsible for the survey, the objectives and content of the survey, voluntary participation and data protection. They provided their informed consent in writing.

## 3. Response rates and representativeness

### 3.1 Comparison of unadjusted and adjusted gross sample

39,247 people (19,044 girls, 20,203 boys) received an invitation to participate in the survey. Of them, 9,230 children and adolescents (4,439 girls, 4,791 boys) were invited to participate in the examination and interview programme (unadjusted gross sample). The composition of both samples was compared regarding the information provided by population registries (age, gender and nationality) or regarding the information from the sample scheme (size of municipalities and regions (West and East German federal states, Berlin)) to ensure the independence of both of the samples drawn. The analysis showed no significant differences between groups.

For the examination and interview group, the share of quality neutral losses was 6.8% and was therefore slightly higher than for the interview group (3.9%). This is primarily owing to the exclusion of people with only rudimentary knowledge of German (see chapter 2.6).

### 3.2 Number of participants and response rates

A total of 15,023 people (7,538 girls, 7,485 boys) participated in KiGGS Wave 2 (total sample, Table 1; stratified by age, see Annex 1). They provide the basis for all analyses referring to interview data. 3,567 children and adolescents (1,801 girls, 1,766 boys) also took part in the examination component. This subgroup participated in medical examinations and tests and provided blood and urine samples. The response rate was 40.1% for the total sample, and with 41.5% slightly higher for the examination subgroup.

The analysis of response rates regarding sociodemographic characteristics revealed the influence of the nationality for both the total gross sample and the examination and interview subgroup. The response rate of study participants with German nationality was significantly higher in the total sample (42.6%) than of those without German nationality (17.0%). However, the examination and interview subgroup managed to attract a higher number of participants without German nationality (response rate 27.9%). Furthermore, the response rates of female participants were generally slightly higher than those of male participants, as well as slightly higher for the former East German than for the West German federal states and Berlin. For nearly all age groups, the response rates ranged between 39.0% and 42.0%, yet were considerably higher in the 7 to 10 year-old examination and interview subgroup (47.0%). Willingness to participate was significantly greater in municipalities with under 20,000 inhabitants than in larger cities for the overall sample and the interview-only subgroup. In the examination subgroup, this pattern was observed for municipalities with up to 100,000 inhabitants.

### 3.3 Composition of the realised sample

The representativeness of the realised sample is assessed by comparing it to data from official statistics (Microcensus 2013). Even though little data is available one key feature can be taken into account regarding the educational distribution. It is related to health parameters and represented by the same indicator (highest parental educational degree according to the CASMIN classification [15]).

**Figure 3:**
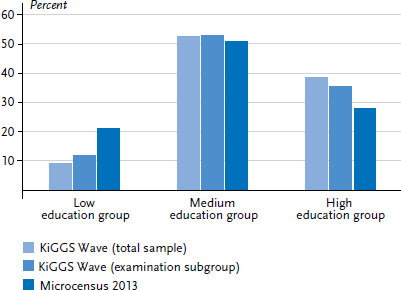
Comparison of highest parental education group of participants and head of household in Microcensus 2013 (total sample n=14,762, examination subgroup n=3,426) Source: KiGGS Wave 2 (2014-2017), Research Data Centres of the Federal Statistical Office and Statistical Offices of the Länder [16]

Figure 3 shows the share of educational level groups in the realised samples. Medium and higher education groups were incorporated commensurate to their share in the population. However, the lower education group, despite the measures aimed at increasing the participation of this group in the survey, has not been reached to the same degree. Weighting factors, however, that close the gap between the realised sample and official statistics largely balance the impact of this deviation on the health parameters surveyed.

## 4. Weighting

The KiGGS Wave 2 cross-sectional sample involved calculating two weighting variables. One variable was applied to the total number of participants, the other to the examination subgroup. Both weighting variables were calculated based on the same proceeding. Weighting was applied both at the level of survey design and an adjustment to account for known population distributions. Weighting at the survey design level affects two probabilities: selection of a particular sample point and selection of participants within the sample point. After this weighting at the survey design level, data was adjusted to account for official population statistics regarding age in years, gender, federal state (as of 31 December 2015) and foreigner status (German nationality yes/no; as of 31 December 2014). Additionally, the distribution of the highest parental education levels according to the CASMIN classification [15] was adapted to match the distribution of head of household education levels surveyed in the Microcensus (2013 [16], limited to households with children under 18 years).

## 5. Discussion and conclusion

Key factors for developing population-based representative information about the health of children and adolescents in Germany are a sufficiently large group of participants and an unbiased composition of the net sample as far as possible. The KiGGS Wave 2 cross-sectional survey does justice to these requirements thanks to several, sometimes complex, measures.

Firstly, an adequate and established method for drawing representative population samples was applied. The selected sample points ensured a representative model of Germany’s settlement structure.

Secondly, a broad set of measures aim to achieve the highest level of participation possible. They were particularly important because filling out the questionnaires and, in particular, the appointments at examination centres, involved a considerable investment of time by participants. Overall, the survey recruited the stipulated number of participants and produced a reliable sample for the target population. Against the backdrop of a widespread decline in willingness among the population to participate in empirical surveys [17, 18], the achieved response rates are satisfactory and comparable to those achieved in other health monitoring surveys (telephone interview survey in KiGGS Wave 1 cross-section (2009-2012): 38.8% [19]; health interview and examination survey for adults DEGS cross-section (2008-2011): 42.0% [20], GEDA 2014/2015-EHIS 26.9% [21]). The measures to recruit participants are not only important in achieving high levels of participation, but also in achieving a composition of the realised sample which is as unbiased as possible [22]. While recruiting participants through telephone calls or visiting them at their homes does cost a lot of time, it is well worth the effort as it increases response rates by so-called “hard-to-reach” groups. The described differences in the composition of the realised total sample and the examination subgroup can be largely put down to the fact that the survey did not have sufficient capacities to approach all potential participants with the same focus (in particular concerning visiting potential participants at their homes) as was the case for the examination and interview group. The higher share of non-German nationality participants, as well as those with low-education backgrounds in the examination and interview group, is owed mainly to this personal contact. The slightly higher willingness to participate in the examination and interview group may also be due to the greater attractiveness of examinations and tests, in particular, because participating children and adolescents received their results individually.

Thirdly, the composition of the realised sample was controlled using known parameters. To compensate for slight biases of the sample with regard to the total population, weighting factors were calculated to analyse survey data.

Overall, the survey showed that continuously monitoring the results of participant recruitment during the process itself is important in order to be able to already have taken adequate measures during the survey. This concerns both ensuring high response rates and sample composition. Against this backdrop, further analyses are planned to discern the degree by which individual measures contributed towards increasing the participation of hard-to-reach segments of the population in health monitoring surveys. It also became clear that the current methods and concepts only inadequately represent those segments of the population with only rudimentary knowledge of German. In future, more needs to be done to overcome language and cultural barriers, both during the recruiting of participants and during interviews and examinations. Feasibility studies are currently testing multilingual interviewers, examination instructions and tele interpretation services [13].

## Key statements

- The KiGGS Wave 2 cross-sectional survey provides up-to-date population-based representative data on the health of children and adolescents in Germany.
- A total of 15,023 children and adolescents from Germany and their parents took part in the cross-sectional KiGGS Wave 2 survey. A response rate of 40.1% was achieved.
- Interview data is available for all children and adolescents. For a subgroup of 3,567 children and adolescents aged at least three years, physical examinations, tests and laboratory analyses were also conducted.
- A set of measures aimed to ensure the greatest number of participants possible and achieve a net sample that reflects as far as possible the composition of the overall population.

## Figures and Tables

**Table 1:** Overview of study participants in KiGGS Wave 2 Source: KiGGS Wave 2 (2014-2017)

	Total sample (Examination and interview + interview group)			Examination subgroup (Examination and interview group)		
	Girls	Boys	Total	Girls	Boys	Total
Initial gross sample	19,044	20,203	39,247	4,439	4,791	9,230
Quality neutral losses	838	989	1,827	288	345	633
Gross sample	18,206	19,214	37,420	4,151	4,446	8,597
Participants	7,538	7,485	15,023	1,801	1,766	3,567
Non-responders	10,668	11,729	22,397	2,350	2,680	5,030
Response rate	41.4%	39.0%	40.1%	43.4%	39.7%	41.5%

## Appendix

**Annex 1:** Net sample according to gender and age Source: KiGGS Wave 2 (2014-2017)

Age	Interview and examination group			Interview group			Total sample		
	Girls	Boys	Total	Girls	Boys	Total	Girls	Boys	Total
0				297	279	576	297	279	576
1				251	227	478	251	227	478
2				180	221	401	180	221	401
3	104	101	205	260	272	532	364	373	737
4	112	123	235	322	361	683	434	484	918
5	97	116	213	333	353	686	430	469	899
6	114	118	232	328	323	651	442	441	883
7	108	114	222	325	341	666	433	455	888
8	112	123	235	335	336	671	447	459	906
9	129	118	247	349	352	701	478	470	948
10	88	119	207	271	312	583	359	431	790
11	141	131	272	392	369	761	533	500	1,033
12	139	140	279	381	370	751	520	510	1,030
13	131	134	265	346	352	698	477	486	963
14	137	118	255	333	359	692	470	477	947
15	127	124	251	386	318	704	513	442	955
16	134	96	230	349	297	646	483	393	876
17	128	91	219	299	277	576	427	368	795
0-2				728	727	1,455	728	727	1,455
3-10	864	932	1,796	2,523	2,650	5,173	3,387	3,582	6,969
11-17	937	834	1,771	2,486	2,342	4,828	3,423	3,176	6,599
Total	1,801	1,766	3,567	5,737	5,719	11,456	7,538	7,485	15,023

## References

[1] Kurth BM Ziese T Tiemann F (2005) Gesundheitsmonitoring auf Bundesebene. Ansätze und Perspektiven. Bundesgesundheitsbl Gesundheitsforsch Gesundheitsschutz 48(3):261-272 10.1007/s00103-004-1001-6 15768298

[2] Kurth BM Lange C Kamtsiuris P (2009) Gesundheitsmonitoring am Robert Koch-Institut. Sachstand und Perspektiven. Bundesgesundheitsbl Gesundheitsforsch Gesundheitsschutz 52(5):557-570 10.1007/s00103-009-0843-3 19343279

[3] Kurth BM Kamtsiuris P Hölling H (2008) The challenge of comprehensively mapping children‘s health in a nation-wide health survey: design of the German KiGGS-Study. BMC Public Health 8(1):196 18533019 10.1186/1471-2458-8-196 PMC2442072

[4] Mauz E Gößwald A Kamtsiuris P (2017) New data for action. Data collection for KiGGS Wave 2 has been completed. Journal of Health Monitoring 2(S3):2-27. http://edoc.rki.de/oa/articles/revpaHQ3DqMU/PDF/25Pxmf2f-cHqRM.pdf (As at 27.09.2017) 10.17886/RKI-GBE-2017-105 PMC10291840 37377941

[5] Lange M Hoffmann R Mauz E (2018) KiGGS Wave 2 longitudinal component – data collection design and developments in the numbers of participants in the KiGGS cohort. Journal of Health Monitoring 3(1):92-107. (As at 15.03.2018) 10.17886/RKI-GBE-2018-035 PMC8848915 35586182

[6] Kurth BM Kamtsiuris P Hölling H (2016) Strategien des Robert Koch-Instituts zum Monitoring der Gesundheit von in Deutschland lebenden Kindern und Jugendlichen. Kinder- und Jugendmedizin 16(3):176-183

[7] Hölling H Schlack R Kamtsiuris P (2012) Die KiGGS-Studie. Bundesweit repräsentative Längs- und Querschnittstudie zur Gesundheit von Kindern und Jugendlichen im Rahmen des Gesundheitsmonitorings am Robert Koch-Institut. Bundesgesundheitsbl Gesundheitsforsch Gesundheitsschutz 55(6-7):836-842 10.1007/s00103-012-1486-3 22736165

[8] Kamtsiuris P Lange M Schaffrath Rosario A (2007) Der Kind-er-und Jugendgesundheitssurvey (KiGGS): Stichprobendesign, Response und Nonresponse-Analyse. Bundesgesundheitsbl Gesundheitsforsch Gesundheitsschutz 50(5):547-556. http://edoc.rki.de/oa/articles/rej53eEjT1Ze6/PDF/211Cul3e7Mh-kk.pdf (As at 26.02.2018) 10.1007/s00103-007-0215-9 17514438

[9] BIK GmbH (2001) BIK Regionen. Ballungsräume, Stadtregionen, Mittel-/Unterzentrengebiete. Methodenbeschreibung zur Aktualisierung 2000. Aschpurwis + Behrens GmbH, Markt-, Mediaund Regionalforschung, Hamburg

[10] Robert Koch-Institut (Ed) (2017) Methodische Studie zur Durchführung von Mixed-Mode-Befragungen zur Gesundheit von Kindern und Jugendlichen (Pilotstudie KiGGS Welle 2). Gesundheitsberichterstattung des Bundes. Gemeinsam getragen von RKI und Destatis. Robert Koch-Institut, Berlin. http://edoc.rki.de/documents/rki_fv/rex2KXJHIPpM/PDF/226m8YvEr7dPM.pdf (As at 26.02.2018)

[11] Hölling H Kamtsiuris P Lange M (2007) Der Kinder- und Jugendgesundheitssurvey (KiGGS): Studienmanagement und Durchführung der Feldarbeit. Bundesgesundheitsbl Gesundheitsforsch Gesundheitsschutz 50(5-6):557-566. http://edoc.rki.de/oa/articles/rej53eEjT1Ze6/PDF/29ruDT0W37I-rU.pdf (As at 26.02.2018) 10.1007/s00103-007-0216-8 17514439

[12] Allen J Schmich P (2016) Gesundheit in Deutschland aktuell (GEDA). Eine repräsentative Gesundheitsbefragung im neuen (Mixed-Mode-) Design. In: Eifler S Faulbaum F (Eds). Methodische Probleme von Mixed-Mode-Ansätzen in der Umfrageforschung. Springer VS, Wiesbaden

[13] Frank LK Yesil-Jürgens R Born S (2018) Improving the inclusion and participation of children and adolescents with a migration background in KiGGS Wave 2. Journal of Health Monitoring 3(1):126-142. www.rki.de/journalhealthmonitoring-en (As at 15.03.2018) 10.17886/RKI-GBE-2018-034 PMC8848790 35586177

[14] The American Association for Public Opinion Research (AAPOR) (2015) Standard Definitions: Final Dispositions of Case Codes and Outcome Rates for Surveys (8th edition). AAPOR

[15] Brauns H Scherer S Steinmann S (2003) The CASMIN Educational Classification in International Comparative Research. In: Hoffmeyer-Zlotnik JHP Wolf C (Eds). Advances in Cross-National Comparison: A European Working Book for Demographic and Socio-Economic Variables. Springer US, Boston, MA, P. 221-244

[16] Research Data Centres of the Federal Statistical Office and Statistical Offices of the Länder (2017) Microcensus,. 2013, own calculations. www.forschungsdatenzentrum.de/bestand/mikrozensus/ (As at 20.11.2017)

[17] Mindell JS Giampaoli S Goesswald A (2015) Sample selection, recruitment and participation rates in health examination surveys in Europe – experience from seven national surveys. BMC Medical Research Methodology 15(78) 10.1186/s12874-015-0072-4 PMC4595185 26438235

[18] Galea S Tracy M (2007) Participation Rates in Epidemiologic Studies. Annals of Epidemiology 17(9):643-653 17553702 10.1016/j.annepidem.2007.03.013

[19] Lange M Butschalowsky H Jentsch F (2014) Die erste KiGGS-Folgebefragung – KiGGS Welle 1. Studiendurchführung, Stichprobendesign und Response. Bundesgesundheitsbl Gesundheitsforsch Gesundheitsschutz 57(7):747-761. http://edoc.rki.de/oa/articles/re5weWnRsXRSw/PDF/20B6fVTP-FIdw.pdf (As at 26.02.2018) 10.1007/s00103-014-1973-9 24950824

[20] Kamtsiuris P Lange M Hoffmann R (2013) Die erste Welle der Studie zur Gesundheit Erwachsener in Deutschland (DEGS1). Stichprobendesign, Response, Gewichtung und Repräsentativität. Bundesgesundheitsbl Gesundheitsforsch Gesundheitsschutz 56(5/6):620-630. http://edoc.rki.de/oa/articles/reOjvEr900Q1Q/PDF/22VmD-7JrO6CNg.pdf (As at 26.02.2018) 10.1007/s00103-012-1650-9 23703478

[21] Saß AC Lange C Finger JD (2017) German Health Update: New data for Germany and Europe. The background to and methodology applied in GEDA 2014/2015-EHIS. Journal of Health Monitoring 2(1):75-82. http://edoc.rki.de/oa/articles/reO6y1z44DhJg/PDF/28vgwL-8wcSGG2.pdf (As at 26.02.2018) 10.17886/RKI-GBE-2017-023 PMC10161278 37151302

[22] Lynn P (2008) The Problem of Nonresponse. In: Leeuw Edith de HJ Dillman Don A (Eds) International Handbook of Survey Methodology. Taylor and Francis, New York, P. 35-55

